# The Bucharest international (ESOT) consensus on controlled donation after circulatory determination of death

**DOI:** 10.3389/ti.2026.17566

**Published:** 2026-08-28

**Authors:** Umberto Cillo, Gabriel C. Oniscu

**Affiliations:** 1 Department of Surgery, Oncology and Gastroenterology (DISCOG), University of Padua, Padua, Italy; 2 Hepato-Bilio-Pancreatic Surgery and Liver Transplantation Unit, Azienda Ospedale Universita Padova, Padua, Italy; 3 Division of Transplantation Surgery, CLINTEC, Karolinska Institutet, Stockholm, Sweden; 4 Department of Transplantation, Karolinska Universitetssjukhuset, Stockholm, Sweden

**Keywords:** consensus, deceased donation, determination of death, donation after circulatory determination of death (DCDD), european society for organ transplantation (ESOT)

Recent clinical-scientific discussions in the field of transplantation have highlighted the need to better shape the Evidence-to-Decision pathway as a continuous dynamic process, translating scientific evidence into concrete clinical decisions/protocols. In response to this need, ESOT has established a Society-Driven Consensus Platform as a permanent framework to ensure that clinical guidance evolves alongside scientific progress. This is particularly challenging in the field of transplantation where there may be a high degree of variability, given the clinical complexity and the relatively low patient numbers that may hamper the robustness of the evidence. Methodologically sound consensuses, as the ones elaborated in the context of the ESOT Consensus Platform, aim to fill this gap and address these challenges, with a 360° perspective, through the involvement of renowned experts worldwide. In this context, the Bucharest initiative represents one of the most paradigmatic consensuses undertaken in the field of transplantation.

Controlled donation after circulatory determination of death (cDCDD) has become the fastest-growing pathway to deceased organ donation and, in some countries, is now the predominant one. Its’ expansion has been driven by advances in machine perfusion, which have transformed the ability to recover, preserve, assess, and potentially repair donor organs. Yet this rapid progress has also exposed important challenges. Clinical outcomes remain heterogeneous, practices differ across centers and countries, and the use of emerging perfusion technologies raise fundamental questions about donation itself, including how death should be determined and how the dead donor rule should be seen and respected in this context. Despite the rapid evolution of the field, international consensus initiatives dedicated specifically to cDCDD have lacked. In this context, the European Society for Organ Transplantation (ESOT) convened the international cDCDD Consensus in Bucharest in October 2024, the results of which are presented in this Special Issue of Transplant International Martin et al.

This effort was deliberately ambitious in both scope and methodology. Four steering committees addressed the adult pathway, the paediatric pathway, normothermic regional perfusion (NRP), and the unifying concept of death. Each committee assembled an international panel of experts and conducted a two-round Delphi process, with statements accepted only after reaching a 75% agreement threshold. The process robustness was further reinforced by an in-person meeting in Bucharest which allowed open discussion of the first-round results and refinement of the statements before the second Delphi round. Overall, around 150 experts from five continents contributed to the project. Coordinating four parallel consensus processes, harmonising their methodology, and integrating the outputs into a coherent framework required a substantial organisational effort, reflecting ESOT’s commitment to fostering global convergence in donation and transplantation and addressing critical and controversial areas [1].

The five contributions are best viewed as complementary components of a single consensus document. At its foundation is the methodology and definitions paper Martin et al. which describes the consensus process but, more importantly, establishes a common language and foundation on which the remaining papers are build and future developments should be constructed. Inconsistent definitions have long hindered meaningful international comparisons. Among the agreed definitions of possible and potential donor, ante-mortem interventions and the critical time-points of the donation pathway, the key one that emerges is the deliberate preference for the term “*donation after circulatory determination of death”* over the broader expression *circulatory death* [2]. We believe that such attention to semantics is transformative when dealing with technically and ethically intricate issues; we therefore advocate a shared vocabulary as an essential prerequisite for collaboration, research, and benchmarking.

Building on this foundation, the adult pathway paper presents thirty-nine recommendations covering system requirements, donor identification, medical suitability, communication, and the management of withdrawal of life-sustaining measures Opdam et al. Two principles, among others, emerge throughout the document. First, the decision to withdraw life-sustaining treatment must remain entirely independent of any consideration of organ donation, thereby preserving the integrity of end-of-life care process. Second, ante-mortem interventions should be evaluated not only for their potential benefit to recipients but also for their ability to respect the donor’s wish to donate, acknowledging the limited evidence currently supporting many of these interventions and highlighting the need for further efforts to produce robust evidence.

The Normothermic Regional Perfusion paper focuses on the technology that reshaped the field, reaching consensus on approximately 130 recommendations Miñambres et al. It provides guidance on team composition and training, equipment, procedural parameters, and the sequence of organ recovery. It also supports the ethical acceptability of NRP within the framework of the dead donor rule, provided cerebral perfusion is reliably prevented. At the same time, it openly acknowledges the issues that remain unresolved. No agreement was reached on the maximum acceptable warm ischaemia time, and important legal differences, particularly regarding thoraco-abdominal NRP and the prevention of cerebral reperfusion that continue to hinder international harmonization.

The paediatric paper is, after the original initiative in Geneva in 2014, the first international structured consensus dedicated to controlled paediatric DCDD and presents forty-seven recommendations Weiss et al. It provides a concrete basis for further local legal and regulatory adaptations to improve global access to paediatric cDCDD. It also addresses the unique challenge posed by children, who very frequently have never expressed their wishes regarding organ donation. The consensus outlines how a competent and sufficiently mature child’s known preferences about donation or end of life should be respected wherever possible. The limited availability of high-quality evidence prompts the need for future research efforts particularly regarding the potential benefits and harms of *ante-mortem* interventions in paediatric cDCDD.

The unifying concept of death paper addresses one of the most challenging issues in cDCDD Procaccio et al. Importantly, consensus was reached on the definition of the unifying brain-based concept of death and the potential benefits of its’ adoption. Further agreements were achieved on the adoption of the concept of permanency rather than irreversibility as the relevant standard when determining death, on a 5-min no-touch period and on the reaffirmation of the dead donor rule as the ethical principle that donation must never cause death. Agreement was achieved on fundamental principles but not on every aspect of their clinical application. The operational definition of death and the clinical criteria required for death determination after the no touch period did not reach the predefined consensus threshold. These unresolved issues are among the most important priorities for future scientific, ethical, and legal debate.

Taken together, the five papers reveal a consistent pattern: strong convergence on fundamental principles and terminology, alongside persistent differences in key areas of clinical practice, including warm ischaemia limits, standards for determining death, and approaches to preventing cerebral perfusion. These differences continue to reflect variations in legal frameworks, healthcare systems, and cultural perspectives across countries. Rather than representing a limitation, these unresolved issues provide a clear agenda for future research, evidence generation, and international harmonization.

Undoubtedly, the Bucharest consensus conference and its outputs represent a milestone in the cDCDD area. However, this should be viewed as an ongoing process and through the ESOT- Consensus Platform, as a permanent expert environment, and the next iteration of the Bucharest consensus will continue to align clinical guidance with rapidly advancing scientific evidence. In the meantime, we are confident that this work will bring international community towards a more consistent clinical and ethical approach to cDCDD, one that embraces scientific innovation while remaining firmly grounded in the fundamental principles of donation and transplantation.
